# Supplementary material for: Variation in floral morphology, histochemistry, and floral visitors of three sympatric morning glory species
Source: PeerJ. 2024 Aug 26;12:e17866. doi: 10.7717/peerj.17866 (PMC11361269; doi:10.7717/peerj.17866)
Supplement: Supplemental Information 1 — Visitation rates of all animal taxa observed at the flowers of Argyreia versicolor, A. mekongensis, and A. lycioides in Sa Kaeo province, Thailand. Animal taxa were categorized as potential pollinators (yellow), visitors/nectar robbers (blue), or florivores (green). [file peerj-12-17866-s001.docx]

**Table S1.** Visitation rates of all animal taxa observed at the flowers of *Argyreia versicolor*, *A. mekongensis*, and *A. lycioides* in Sa Kaeo province, Thailand. Animal taxa were categorized as potential pollinators (yellow), visitors/nectar robbers (blue), or florivores (green).

| **Animal visitors** | **Visitation rates (visits per hour; mean ± SE)** | | | | | |
| --- | --- | --- | --- | --- | --- | --- |
|  | ***A. versicolor*** | | ***A. mekongensis*** | | ***A. lycioides*** | |
|  | **2019** | **2020** | **2019** | **2020** | **2019** | **2020** |
| ***Xylocopa aestuans*** | - | 0.1087 ± 0.0539 | 0.3464 ± 0.162 | 0.497 ± 0.252 | - | - |
| ***Xylocopa latipes*** | 1.6707 ± 0.3989 | 0.405 ± 0.1047 | 0.3873 ± 0.1652 | 0.3868 ± 0.2337 | - | - |
| **Vespidae** | - | - | - | 0.0108 ± 0.0108 | - | 0.1424 ± 0.0882 |
| **Anthophila** | 0.0021 ± 0.0021 | - | - | 0.0270 ± 0.0270 | 0.0244 ± 0.0182 | - |
| ***Amegilla* sp.** | - | - | 0.266 ± 0.1383 | - | - | - |
| **Orthoptera** | - | - | 0.0348 ± 0.0222 | 0.034 ± 0.0277 | - | - |
| ***Cinnyris jugularis*** | - | - | - | 0.0108 ± 0.0108 | - | 0.0814 ± 0.0367 |
| **Hesperiidae** | - | - | - | 0.01 ± 0.01 | - | 0.0029 ± 0.0029 |
| **Blattodea** | - | - | - | - | - | 0.0147 ± 0.0147 |
| **Formicidae** | - | - | - | - | 0.0498 ± 0.0244 | - |
| ***Mylabris phalerata*** | - | - | - | 0.1029 ± 0.0655 | - | - |
