# Supplementary material for: Variation in floral morphology, histochemistry, and floral visitors of three sympatric morning glory species
Source: PeerJ. 2024 Aug 26;12:e17866. doi: 10.7717/peerj.17866 (PMC11361269; doi:10.7717/peerj.17866)
Supplement: Supplemental Information 2 [file peerj-12-17866-s002.docx]

**"Variation in floral morphology, histochemistry, and floral visitors of three sympatric morning glory species" (#2024:03:98554:0:1:REVIEW)**

Dear Editor,

We are very grateful to you and the three reviewers for the feedback on our manuscript. Please see our detailed response to reviewer comments, as well as our revised manuscript with all amendments.

Regarding the addition of the newly added author (Natthaphong Chitchak), some floral data were requested by the reviewers, which we did not measure in our initial study. Dr. Chitchak happened to have this data, which he was happy to share with us (Table 1). Moreover, he performed the statistical analysis on the floral morphology and trichome data, constructed a graph (the newly added Figure 2), and contributed to revising the manuscript, in addition to previously helping the first author with fieldwork and providing advice about trichomes during lab work. Given his valuable contributions to our revised manuscript, we felt that he deserved more than a mere mention in the acknowledgments section (as in our initial submission) and therefore request that he be included as a co-author.

[Note: All current coauthors have already agreed with the change by responding to the change confirmation email].

On behalf of all authors,

Dr. Paweena Traiperm
